# Dietary exposures and allergy prevention in high-risk infants: a joint position statement of the Canadian Society of Allergy and Clinical Immunology and the Canadian Paediatric Society

**DOI:** 10.1186/1710-1492-10-45

**Published:** 2014-09-02

**Authors:** Edmond S Chan, Carl Cummings, Adelle Atkinson, Zave Chad, Marie-Josée Francoeur, Linda Kirste, Douglas Mack, Marie-Noël Primeau, Timothy K Vander Leek, Wade TA Watson

**Affiliations:** BC Children’s Hospital, Department of Pediatrics, Division of Allergy & Immunology, University of British Columbia, Room 1C31B, 4480 Oak St, Vancouver, BC V6H 3V4 Canada; Montreal Children’s Hospital, McGill University Health Centre, Montreal, Quebec Canada; The Hospital for Sick Children, Division of Allergy and Clinical Immunology, Department of Paediatrics, University of Toronto, Toronto, ON Canada; Department of Pediatrics, University of Ottawa, Ottawa, ON Canada; Hôpital Charles LeMoyne, Département de pédiatrie, Service d’allergie et immunologie clinique, Université de Sherbrooke, Greenfield Park, Québec, Canada; Allergy Nutrition Service, Dietitian Services, HealthLinkBC, Burnaby, BC Canada; McMaster University, Hamilton, Ontario Canada; Montreal Children’s Hospital, Department of Pediatrics, Division of Allergy and Immunology, McGill University Health Centre, Montreal, QC Canada; Department of Pediatrics, Faculty of Medicine & Dentistry, University of Alberta, Edmonton, AB Canada; Department of Pediatrics, Dalhousie University, Division of Allergy, IWK Health Centre, Halifax, NS Canada

**Keywords:** Allergy prevention, Atopic dermatitis, Breastfeeding, Food allergy, Formula feeding, Solid food introduction

## Abstract

Allergic conditions in children are a prevalent health concern in Canada. The burden of disease and the societal costs of proper diagnosis and management are considerable, making the primary prevention of allergic conditions a desirable health care objective. This position statement reviews current evidence on dietary exposures and allergy prevention in infants at high risk of developing allergic conditions. It revisits previous dietary recommendations for pregnancy, breastfeeding and formula-feeding, and provides an approach for introducing solid foods to high-risk infants. While there is no evidence that delaying the introduction of any specific food beyond six months of age helps to prevent allergy, the protective effect of early introduction of potentially allergenic foods (at four to six months) remains under investigation. Recent research appears to suggest that regularly ingesting a new, potentially allergenic food may be as important as when that food is first introduced. This article has already been published (Paediatr Child Health. 2013 Dec;18(10):545–54), and is being re-published with permission from the original publisher, the Canadian Paediatric Society.

## Background

The prevalence of food allergy in Canada, based on self-reported data, is estimated to be approximately 7%, a number sufficient to make primary prevention a desirable health care goal [1]. Some recent evidence, notably from Australia, suggests that the prevalence of food allergy in infants is increasing, with over 10% of one-year-olds having an immunoglobulin (Ig)-E-mediated food allergy proven by oral challenges [2]. For years, published guidelines in the United States, Europe and Australia have examined the relationship between allergy prevention in high-risk infants and dietary exposures during pregnancy, lactation and early infancy. The American Academy of Pediatrics changed its advice for allergy prevention in 2008, [3] realigning their recommendations to be consistent with recent European and Australian positions [4–6]. The present statement encapsulates the most important changes to these guidelines for Canadian health care providers, who may not be fully aware of them, and complements current beliefs regarding allergy prevention in the United States, Europe and Australia.

The primary objective of this statement is to inform health care practitioners that best evidence now suggests there is *no* benefit to delaying the introduction of any specific solid food, including highly allergenic proteins, beyond six months of age to prevent food allergy from developing. It advises against restricting maternal diet during pregnancy and lactation, affirms the immunological role of breastfeeding, and offers some guidance on the choice of formula for mothers who cannot or choose not to breastfeed. The recommendations in this statement do not apply to infants with an established food allergy.

### Defining risk

An infant at high risk of developing allergy usually has a first-degree relative (at least one parent or sibling) with an allergic condition such as atopic dermatitis, a food allergy, asthma or allergic rhinitis [7]. This defining relationship corresponds with other published guidelines. While recommendations in the present statement are intended for high-risk infants, some of the studies cited below included infants from the general population who were not considered to be at high risk for developing allergy.

### Maternal diet and breastfeeding duration

Evidence to support maternal dietary restrictions during pregnancy is contradictory and insufficient to change best practice [3–6]. A recent Cochrane review found little evidence that avoiding milk, egg or other potential allergens during pregnancy reduced the risk of atopic eczema or asthma in infants [8]. Recent reports investigating the effect of ingesting peanut during pregnancy were also inconclusive, with authors providing only cautious interpretation of study results because randomized, prospective data were lacking [9]. Similarly, restricting the maternal diet while breastfeeding has not been proven to prevent allergic conditions in infants, with the possible exception of atopic eczema. Higher quality studies are needed [8]. However, while the evidence that restricting maternal diet during pregnancy and lactation helps to prevent allergy is weak, the risks of maternal undernutrition and potential harm to the infant from avoiding these foods may be significant.

Health Canada, the Canadian Paediatric Society (CPS), the Dietitians of Canada and the Breastfeeding Committee of Canada have reiterated the manifest health benefits of exclusive breastfeeding for the first six months of life – which include immunological protection – in a recent position statement [10]. However, the role of breastfeeding in preventing allergy remains unclear because previous studies of the relationship have been insufficiently rigorous or lacked ideal methodological design. To date, studies have been largely observational, non-uniform in terms of breastfeeding duration, and too variable in the diagnostic criteria for allergic conditions [11]. There is some evidence that in infants at high risk of allergy, exclusive breastfeeding for at least the first four months of life is associated with decreased prevalence of atopic dermatitis and cow’s milk allergy during childhood [12]. However, a more recent study has failed to show a protective effect of breastfeeding on atopic dermatitis [13]. There may be evidence of decreased wheezing before four years of age with at least three months of breastfeeding, [14] but there is either no effect or possibly even an increased risk of asthma after six years of age associated with a similar duration of breastfeeding [15].

Some allergy prevention guidelines favour four to six months of exclusive breastfeeding, compared with the WHO’s recommendation of six months, because of potential benefit from tolerance to solid foods during a theoretical “window of opportunity” between four and six months of age [3, 4, 6]. Also, there is a lack of evidence supporting the benefit of avoiding solid foods beyond four months of age to prevent allergy. Some specialists have proposed a compromise: starting to introduce solid foods at four months of age while continuing to breastfeed until an infant is at least six months of age [16]. Working with this compromise may depend on weighing an infant’s unique atopic risk factors against the many benefits of exclusive breastfeeding for six months. A recent randomized study has suggested that introducing solid foods at four months of age while maintaining breastfeeding for at least six months has no impact on growth and improves iron status [17]. Furthermore, one recent prospective cohort study showed that introducing solid foods before six months of age was associated with decreased asthma, allergic rhinitis and atopic sensitization at five years of age [18]. The same study reported that the total duration of breastfeeding was more important for preventing these allergic conditions than exclusive breastfeeding. More research needs to be conducted to confirm these findings. An article published in 2013 by the American Academy of Allergy, Asthma and Immunology has similarly focused on breastfeeding for four to six months of age and the introduction of complementary foods during the same interval in preventing allergy [19].

### Choice of formula

All current recommendations favour breastfeeding over formula-feeding. There have been no long-term studies comparing exclusive breastfeeding with formula-feeding in allergy prevention because it would be unethical to randomize infants to breast- or formula-feeding. Therefore, only different infant formulas can be compared in relation to allergy development.

There is limited evidence to suggest that childhood allergy can be prevented in high-risk infants by using certain hydrolyzed cow’s milk formulas in the first four to six months of life, compared with intact cow’s milk formula [20]. Of the hydrolyzed formulas, extensively hydrolyzed casein formula is more likely to be effective in preventing atopic dermatitis in high-risk infants than partially hydrolyzed whey formula [21–23]. Some formula studies were industry-sponsored.

One recent prospective study suggested that introducing cow’s milk formula to supplement breastfeeding during the first 14 days of life, followed by regular, daily supplementation of breastfeeding with the same formula, may help to prevent cow’s milk allergy by developing tolerance mechanisms [24]. More research is needed to confirm this observation. Studies have suggested an association between lower incidence of atopic dermatitis and the use of hydrolyzed formulas compared with intact cow’s milk formulas, so it is unclear how the potential benefit from early cow’s milk formula supplementation may affect best practice in future. Introducing cow’s milk formula this early would, at the very least, contradict current recommendations from Health Canada, the CPS and the WHO to breastfeed exclusively for the first six months of life for overall health benefit.

No studies have examined the role of amino-acid formulas in allergy prevention, and there is consensus in the literature that soy formula does *not* have a role [3]. It is unclear whether formula-feeding in early infancy has any role in preventing allergic conditions other than atopic dermatitis, and also unclear whether formula-feeding has any preventive effect long-term. No clear recommendations for choice of formula can be made, given the lack of conclusive evidence related to allergy prevention.

### Introducing solid foods

In 2000, the American Academy of Pediatrics recommended delaying the introduction of potential ‘trigger’ foods (eg, cow’s milk protein until one year of age, egg until two years of age, and peanut or seafood until 3 years of age) for infants at high risk of developing allergy [25]. This advice was based on expert opinion because there were no convincing data to support this position at the time.

Since 2000, however, accumulating observational evidence has suggested that delaying the introduction of certain foods does not prevent food allergy; rather, it may actually promote allergy development [26]. There is increasing speculation that a later introduction of peanut has increased the prevalence of peanut allergy. One study in the United Kingdom showed that the prevalence of peanut allergy tripled during the period when public health practitioners were advising parents to delay peanut introduction [27]. Another observational study reported the prevalence of peanut allergy among Jewish children living in Israel at one-tenth that of Jewish children living in the U.K. Most infants in Israel were ingesting peanut protein during the first year of life, while infants born in the U.K. consumed almost none [28]. An American study has suggested that delaying the introduction of wheat and cereal grains beyond six months of age increases the subsequent risk of wheat allergy [29].

A “dual-allergen-exposure hypothesis” has been proposed to explain why delaying the introduction of specific solid foods has contributed to the rise in food allergy incidence [30]. The hypothesis suggests that infants with eczema as an initial risk factor have increased environmental exposure to food topically, via “broken” skin, a sensitizing route of exposure. Delaying introduction to the same food does not permit the infant to derive potential benefits from regular oral and gastrointestinal exposure, or to develop tolerance via regulatory T-cell pathways. In support of sensitization via the skin, a recent study has shown that mutations in filaggrin, a gene key to maintenance of the skin barrier, are a risk factor for IgE-mediated peanut allergy [31]. Another study has suggested that infants with a high environmental exposure to peanut but no oral exposure are at increased risk of developing peanut allergy [32].

The American Academy of Pediatrics issued a new guideline in 2008 that concluded there was no convincing evidence that delaying the introduction of solid foods, including peanut, egg and fish, beyond four to six months of age has a significant protective effect on allergy prevention [3]. Their conclusion was consistent with recommendations from the European Academy of Allergology and Clinical Immunology, the European Society for Pediatric Gastroenterology, Hepatology and Nutrition, and the Australasian Society of Clinical Immunology and Allergy [4–6].

Whether an earlier introduction of solid foods (eg, at or before four to six months of age) has a truly preventative effect also remains to be established. However, emerging data suggests that it is possible. A 2006 Swedish study of a cohort of 4089 infants concluded that regular fish consumption in the first year of life was associated with lower risk of allergic disease (OR 0.76; 95% CI, 0.61-0.94) and reduced allergic sensitization for the first four years of life [33]. A recent cross-sectional study from Australia demonstrated that delaying egg introduction for the first year of life resulted in a 3.4-fold higher risk of developing egg allergy compared with egg introduction at any time between four and six months of age [34]. The LEAP (Learning Early About Peanut) allergy study has been underway for several years in the United Kingdom. This is a prospective trial randomly assigning high-risk infants to either an early introduction of peanut protein (at four to 10 months of age) or delayed introduction (until three years of age), with ongoing exposures of three times per week once introduced [35]. When the study cohort reaches five years of age, there will be evaluation of the presence or absence of peanut allergy using oral challenges. The same investigators have created the EAT (Enquiring About Tolerance) study to determine whether even earlier introduction of specific foods decreases the risk of food allergy. Their prospective trial randomly assigned unselected infants with no increased risk of allergy to regular consumption (ie, several times per week) of allergenic foods (eg, cow’s milk, egg, peanut, fish, sesame, wheat) starting at either three or six months of age [36]. The stipulation for regular ingestion is based on studies examining mechanisms of oral tolerance. It appears that early introduction alone is insufficient to achieve a preventive effect; regular exposure is equally important [30]. Results from the LEAP and EAT studies are expected within the next few years.

Until the results of LEAP, EAT and other clinical trials become available, uncertainty remains. Deciding whether to introduce potentially allergenic solid foods to high-risk infants early should be individualized and based on parental comfort level. The choice of using an older ‘delayed introduction’ approach, with no proven benefit and, possibly, an increased risk of food allergy, versus an unproven ‘early introduction’ approach, whose potential benefits are based on preliminary data, is difficult for clinicians to make. Other factors, such as having an older sibling with peanut allergy or parental reluctance to introduce peanut until testing by a certified allergist has occurred, may complicate decision-making. It is worth noting that routine screening for allergy to a food, using a skin test or specific IgE blood test, without a history of the child ever ingesting the food in question, is generally discouraged. The high risk of false positive results can be confusing [19]. For the family reluctant to introduce a food because of family history or for other reasons, referral to a certified allergist is a better course. A specialist can decide whether an oral food challenge is warranted. Regardless of timing, once a new food is introduced by parents, it appears prudent to recommend regular exposures (eg, several times a week and with a soft mashed consistency to avoid risk of choking) to maintain oral tolerance.

## Conclusions

There has been a recent shift in evidence-based practice to prevent food allergy in high-risk infants. Delaying the introduction of certain ‘trigger’ foods for periods previously recommended has been shown to have *no* protective effect on allergic sensitization and disease development. While the benefits of introducing these foods to infants earlier, at four to six months of age, are yet to be determined, the immunological mechanisms of sensitization and tolerance tend to support the latter approach. Regardless of the optimal timing for introduction, current understanding of immunological tolerance also appears to suggest that regular, frequent oral consumption may be just as important as when a food is first introduced.

### Recommendations

Based on current evidence, and always conceding that much more research needs to be done, the Canadian Society of Allergy and Clinical Immunology and the Canadian Paediatric Society recommend the following approach to prevent allergy in infants who have a first-degree relative with an allergic condition, and are, therefore, considered to be high risk. The levels of evidence reported in the recommendations have been described using the evaluation of evidence criteria out lined by the Canadian Task Force on Preventive Health Care [37].

- Do not restrict maternal diet during pregnancy or lactation. There is no evidence that avoiding milk, egg, peanut or other potential allergens during pregnancy helps to prevent allergy, while the risks of maternal undernutrition and potential harm to the infant may be significant. (Evidence II-2B)
- Breastfeed exclusively for the first six months of life. Whether breastfeeding prevents allergy as well as providing optimal infant nutrition and other manifest benefits is not known. The total duration of breastfeeding (at least six months) may be more protective than exclusive breastfeeding for six months. (Evidence II-2B)
- Choose a hydrolyzed cow’s milk-based formula, if necessary. For mothers who cannot or choose not to breastfeed, there is limited evidence that hydrolyzed cow’s milk formula has a preventive effect against atopic dermatitis compared with intact cow’s milk formula. Extensively hydrolyzed casein formula is likely to be more effective than partially hydrolyzed whey formula in preventing atopic dermatitis. Amino acid-based formula has not been studied for allergy prevention, and there is no role for soy formula in allergy prevention. It is unclear whether any infant formula has a protective effect for allergic conditions other than atopic dermatitis. (Evidence IB)
- Do not delay the introduction of any specific solid food beyond six months of age. Later introduction of peanut, fish or egg does not prevent, and may even increase, the risk of developing food allergy. (Evidence II-2B)
- More research is needed on the early introduction of specific foods to prevent allergy. Inducing tolerance by introducing solid foods at four to six months of age is currently under investigation and cannot be recommended at this time. The benefits of this approach need to be confirmed by a rigorous prospective trial. (Evidence II-2B)
- Current research on immunological responses appears to suggest that the regular ingestion of newly introduced foods (eg, several times a week and with a soft mashed consistency to prevent choking) is important to maintain tolerance. However, routine skin or specific IgE blood testing before a first ingestion is discouraged, due to the high risk of potentially confusing false positive results. (Evidence II-2B)

### Resource for families

- HealthLink BC, 2013. Reducing risk of food allergy in your baby: A resource for parents of babies at increased risk of food allergy. Endorsed by the Canadian Society of Allergy and Clinical Immunology: http://www.healthlinkbc.ca/healthyeating/reducing-food-allergy-baby.html

## References

[1] Soller L, Ben-Shoshan M, Harrington DW, Fragapane J, Joseph L, St Pierre Y, Godefroy SB, La Vieille S, Elliott SJ, Clarke AE (2012). Overall prevalence of self-reported food allergy in Canada. J Allergy Clin Immunol.

[2] Osborne NJ, Koplin JJ, Martin PE, Gurrin LC, Lowe AJ, Matheson MC, Ponsonby AL, Wake M, Tang ML, Dharmage SC, Allen KJ (2011). Prevalence of challenge-proven IgE-mediated food allergy using population-based sampling and predetermined challenge criteria in infants. J Allergy Clin Immunol.

[3] Greer FR, Sicherer SH, Burks AW, American Academy of Pediatrics Committee on Nutrition; American Academy of Pediatrics Section on Gastroenterology, Hepatology, and Nutrition (2008). Effects of early nutritional interventions on the development of atopic disease in infants and children: The role of maternal dietary restriction, breastfeeding, timing of introduction of complementary foods, and hydrolyzed formulas. Pediatrics.

[4] Høst A, Halken S, Muraro A, Dreborg S, Niggemann B, Aalberse R, Arshad SH, von Berg A, Carlsen KH, Duschén K, Eigenmann PA, Hill D, Jones C, Mellon M, Oldeus G, Oranje A, Pascual C, Prescott S, Sampson H, Svartengren M, Wahn U, Warner JA, Warner JO, Vandenplas Y, Wickman M, Zeiger RS (2008). Dietary prevention of allergic diseases in infants and small children. Pediatr Allergy Immunol.

[5] Agostoni C, Decsi T, Fewtrell M, Goulet O, Kolacek S, Koletzko B, Michaelsen KF, Moreno L, Puntis J, Rigo J, Shamir R, Szajewska H, Turck D, van Goudoever J, ESPGHAN Committee on Nutrition (2008). Complementary feeding: a commentary by the ESPGHAN Committee on Nutrition. J Pediatr Gastroenterol Nutr.

[6] Prescott SL, Tang ML, Australasian Society of Clinical Immunology and Allergy (2005). The Australasian Society of Clinical Immunology and Allergy position statement: Summary of allergy prevention in children. Med J Aust.

[7] Muraro A, Dreborg S, Halken S, Høst A, Niggemann B, Aalberse R, Arshad SH, von Berg A, Carlsen KH, Duschén K, Eigenmann P, Hill D, Jones C, Mellon M, Oldeus G, Oranje A, Pascual C, Prescott S, Sampson H, Svartengren M, Vandenplas Y, Wahn U, Warner JA, Warner JO, Wickman M, Zeiger RS (2004). Dietary prevention of allergic diseases in infants and small children. Part II. Evaluation of methods in allergy prevention studies and sensitization markers. Definitions and diagnostic criteria of allergic diseases. Pediatr Allergy Immunol.

[8] Kramer MS, Kakuma R (2012). Maternal dietary antigen avoidance during pregnancy or lactation, or both, for preventing or treating atopic disease in the child. Cochrane Database Syst Rev.

[9] Sicherer SH, Wood RA, Stablein D, Lindblad R, Burks AW, Liu AH, Jones SM, Fleischer DM, Leung DY, Sampson HA (2011). Maternal consumption of peanut during pregnancy is associated with peanut sensitization in atopic infants. J Allergy Clin Immunol.

[10] Health Canada (2012). Nutrition for Healthy Term Infants: Recommendations from Birth to Six Months. A joint statement of Health Canada, the Canadian Paediatric Society, the Dietitians of Canada, and the Breastfeeding Committee for Canada.

[11] Kramer MS (2011). Breastfeeding and allergy: the evidence. Ann Nutr Metab.

[12] Gdalevich M, Mimouni D, David M, Mimouni M (2001). Breast-feeding and the onset of atopic dermatitis in childhood: a systematic review and meta-analysis of prospective studies. J Am Acad Dermatol.

[13] Flohr C, Nagel G, Weinmayr G, Kleiner A, Strachan DP, Williams HC, ISAAC Phase Two Study Group (2011). Lack of evidence for a protective effect of prolonged breastfeeding on childhood eczema: lessons from the International Study of Asthma and Allergies in Childhood (ISAAC) Phase Two. Br J Dermatol.

[14] Kull I, Almqvist C, Lilja G, Pershagen G, Wickman M (2004). Breast-feeding reduces the risk of asthma during the first 4 years of life. J Allergy Clin Immunol.

[15] Sears MR, Greene JM, Willan AR, Taylor DR, Flannery EM, Cowan JO, Herbison GP, Poulton R (2002). Long-term relation between breastfeeding and development of atopy and asthma in children and young adults: a longitudinal study. Lancet.

[16] Prescott SL, Smith P, Tang M, Palmer DJ, Sinn J, Huntley SJ, Cormack B, Heine RG, Gibson RA, Makrides M (2008). The importance of early complementary feeding in the development of oral tolerance: Concerns and controversies. Pediatr Allergy Immunol.

[17] Jonsdottir OH, Thorsdottir I, Hibberd PL, Fewtrell MS, Wells JC, Palsson GI, Lucas A, Gunnlaugsson G, Kleinman RE (2012). Timing of the introduction of complementary foods in infancy: a randomized controlled trial. Pediatrics.

[18] Nwaru BI, Takkinen HM, Niemelä O, Kaila M, Erkkola M, Ahonen S, Haapala AM, Kenward MG, Pekkanen J, Lahesmaa R, Kere J, Simell O, Veijola R, Ilonen J, Hyöty H, Knip M, Virtanen SM (2013). Timing of infant feeding in relation to childhood asthma and allergic diseases. J Allergy Clin Immunol.

[19] Fleischer DM, Spergel JM, Assa’ad AH, Pongracic JA (2013). Primary prevention of allergic disease through nutritional interventions. J Allergy Clin Immunol.

[20] Osborn DA, Sinn J (2006). Formulas containing hydrolysed protein for prevention of allergy and food intolerance in infants. Cochrane Database Syst Rev.

[21] von Berg A, Filipiak-Pittroff B, Krämer U, Hoffmann B, Link E, Beckmann C, Hoffmann U, Reinhardt D, Grübl A, Heinrich J, Wichmann HE, Bauer CP, Koletzko S, Berdel D, GINIplus study group (2013). Allergies in high-risk schoolchildren after early intervention with cow’s milk protein hydrolysates:10-year results from the German Infant Nutritional Intervention (GINI) study. J Allergy Clin Immunol.

[22] Lowe AJ, Hosking CS, Bennett CM, Allen KJ, Axelrad C, Carlin JB, Abramson MJ, Dharmage SC, Hill DJ (2011). Effect of a partially hydrolyzed whey infant formula at weaning on risk of allergic disease in high-risk children: a randomized controlled trial. J Allergy Clin Immunol.

[23] Chung CS, Yamini S, Trumbo PR (2012). FDA’s health claim review: whey-protein partially hydrolyzed infant formula and atopic dermatitis. Pediatrics.

[24] Katz Y, Rajuan N, Goldberg MR, Eisenberg E, Heyman E, Cohen A, Leshno M (2010). Early exposure to cow’s milk protein is protective against IgE-mediated cow’s milk protein allergy. J Allergy Clin Immunol.

[25] American Academy of Pediatrics. Committee on Nutrition (2000). Hypoallergenic infant formulas. Pediatrics.

[26] Nwaru BI, Takkinen HM, Niemelä O, Kaila M, Erkkola M, Ahonen S, Tuomi H, Haapala AM, Kenward MG, Pekkanen J, Lahesmaa R, Kere J, Simell O, Veijola R, Ilonen J, Hyöty H, Knip M, Virtanen SM (2013). Introduction of complementary foods in infancy and atopic sensitization at the age of 5 years: Timing and food diversity in a Finnish birth cohort. Allergy.

[27] Hourihane JO, Aiken R, Briggs R, Gudgeon LA, Grimshaw KE, Dunn Galvin A, Roberts SR (2007). The impact of government advice to pregnant mothers regarding peanut avoidance on the prevalence of peanut allergy in United Kingdom children at school entry. J Allergy Clin Immunol.

[28] Du Toit G, Katz Y, Sasieni P, Mesher D, Maleki SJ, Fisher HR, Fox AT, Turcanu V, Amir T, Zadik-Mnuhin G, Cohen A, Livne I, Lack G (2008). Early consumption of peanuts in infancy is associated with a low prevalence of peanut allergy. J Allergy Clin Immunol.

[29] Poole JA, Barriga K, Leung DY, Hoffman M, Eisenbarth GS, Rewers M, Norris JM (2006). Timing of initial exposure to cereal grains and the risk of wheat allergy. Pediatrics.

[30] Lack G (2012). Update on risk factors for food allergy. J Allergy Clin Immunol.

[31] Brown SJ, Asai Y, Cordell HJ, Campbell LE, Zhao Y, Liao H, Northstone K, Henderson J, Alizadehfar R, Ben-Shoshan M, Morgan K, Roberts G, Masthoff LJ, Pasmans SG, van den Akker PC, Wijmenga C, Hourihane JO, Palmer CN, Lack G, Clarke A, Hull PR, Irvine AD, McLean WH (2011). Loss-of-function variants in the filaggrin gene are a significant risk factor for peanut allergy. J Allergy Clin Immunol.

[32] Fox AT, Sasieni P, du Toit G, Syed H, Lack G (2009). Household peanut consumption as a risk factor for the development of peanut allergy. J Allergy Clin Immunol.

[33] Kull I, Bergström A, Lilja G, Pershagen G, Wickman M (2006). Fish consumption during the first year of life and development of allergic diseases during childhood. Allergy.

[34] Koplin JJ, Osborne NJ, Wake M, Martin PE, Gurrin LC, Robinson MN, Tey D, Slaa M, Thiele L, Miles L, Anderson D, Tan T, Dang TD, Hill DJ, Lowe AJ, Matheson MC, Ponsonby AL, Tang ML, Dharmage SC, Allen KJ (2010). Can early introduction of egg prevent egg allergy in infants? A population-based study. J Allergy Clin Immunol.

[35] Du Toit G, Roberts G, Sayre PH, Plaut M, Bahnson HT, Mitchell H, Radulovic S, Chan S, Fox A, Turcanu V, Lack G, Learning Early About Peanut Allergy (LEAP) Study Team (2013). Identifying infants at high risk of peanut allergy: the Learning Early about Peanut Allergy (LEAP) screening study. J Allergy Clin Immunol.

[36] **EAT Study (Enquiring About Tolerance)** (Accessed June 25, 2014) http://www.eatstudy.co.uk

[37] Canadian Task Force on Preventive Health Care (2003). New grades for recommendations from the Canadian Task Force on Preventive Health Care for specific clinical preventive actions. CMAJ.

